# Genomic diversity of resistant and virulent factors of *Burkholderia pseudomallei* clinical strains recovered from Guangdong using whole genome sequencing

**DOI:** 10.3389/fmicb.2022.980525

**Published:** 2022-10-28

**Authors:** Muhammad Shafiq, Bixia Ke, Xin Li, Mi Zeng, Yumeng Yuan, Dongmei He, Xiaoling Deng, Xiaoyang Jiao

**Affiliations:** ^1^Department of Cell Biology and Genetics, Shantou University Medical College, Shantou, China; ^2^Center for Disease Control and Prevention of Guangdong Province, Chinese Academy of Sciences, Guangzhou, China

**Keywords:** *Burkholderia pseudomallei*, melioidosis, resistance, virulence, genomic diversity

## Abstract

**Background:**

*Burkholderia pseudomallei* (*B. pseudomallei*) is a highly infectious agent and causes melioidosis, in both humans and animals, which is endemic in Southeast Asia and Northern Australia.

**Objectives:**

This study aims to determine the molecular epidemiology, resistant determinants, and genomic diversity of the clinical isolates of *B. pseudomallei* to further elucidate the phylogenetic and evolutionary relationship of the strains with those in other endemic regions.

**Methods:**

In this study, we obtained eight clinical *B. pseudomallei* isolates from Guangdong province from 2018 to 2019. All the isolates were sequenced using the Illumina NovaSeq platform. The draft genomes of *B. pseudomallei* were further used to find antibiotic-resistant genes (ARGs), virulence factors, and gene mutations. Multilocus sequence typing (MLST) and single nucleotide polymorphism (SNP) analysis were performed to characterize the diversity and epidemiology of the strains.

**Results:**

All isolates were susceptible to antibiotics commonly used for melioidosis treatment. Class D beta-lactamases genes OXA-57 and OXA-59, as well as various mutation factors such as *amrA*, *amrB*, *omp38*, *gyrA*, and *ceoB* were identified. MLST analysis of the *B. pseudomallei* strains identified eight different sequence types (STs): ST1774, ST1775, ST271, ST562, ST46, ST830, ST1325, and ST10. Phylogenetic analysis found that the strains used in this study showed high genetic diversity. We also report 165 virulence factors among *B. pseudomallei* strains responsible for different neurological disorders, pneumonia, skin lesions, and abscesses. All strains recovered in this study were susceptible to commonly used antibiotics. However, high genetic diversity exists among the isolates. The surveillance, diagnosis, and clinical features of melioidosis varied in different geographical locations. These regional differences in the clinical manifestations have implications for the practical management of the disease.

**Conclusion:**

The present study reports the identification of different mutation and virulence factors among *B. pseudomallei* strains responsible for different neurological disorders, pneumonia, skin lesions, and abscesses.

## Introduction

*B. pseudomallei* is a highly pathogenic Gram-negative bacterium and is the etiologic agent of melioidosis, a life-threatening zoonotic infectious disease of tropical and subtropical areas mainly in Northern Australia and Southeast Asia. Melioidosis was first recognized in 1911 (Whitmore, 1913) as an endemic infectious disease, with high pathogenicity, that is challenging to diagnose and treat due to its diverse clinical manifestations. The clinical manifestations can be latent, acute, or chronic (according to disease course), and the patients who develop clinical symptoms are considered to have melioidosis. Of the melioidosis cases, 85% result in acute infections and 11% result in chronic melioidosis that lasts >2 months, while 5% result in latent infection that shows asymptomatic symptoms (Currie et al., 2010). People are mainly infected through contaminated water and soil and damaged skin (Limmathurotsakul et al., 2016).

Recent phylogenomic analysis using whole-genome sequences of 469 *B. pseudomallei* isolates, collected from humans and the environment, from 30 countries collected over 79 years revealed that the ancestors of *B. pseudomallei* first arose in Australia and then was transmitted to South Asia and East Asia (Chewapreecha et al., 2017). Another modeling study in 2016 estimated that melioidosis cases are rising worldwide, highlighting about 46 countries as endemic areas, with ~165,000 confirmed cases and 89,000 (54%) human deaths annually (Limmathurotsakul et al., 2016). A wide range of underlying conditions predispose individuals to melioidosis, but the most common risk factor is diabetes mellitus, which is present in >50% of all patients with melioidosis (Wiersinga et al., 2018). More than 80% of diabetics live in low-income and middle-income countries, and the percentage is expected to rise by >55% globally by 2050 (Currie et al., 2004). The Center for Disease Control and Prevention (CDC) has classified melioidosis as a Category I and Category B biothreat agent (Centers for Disease Control and Prevention (CDC), 2012; Chen and Li, 2018). Although it has been more than 100 years since the discovery of melioidosis, people generally lack a complete understanding of the disease because of its diverse clinical symptoms, fault diagnosis, and high mortality. No vaccine is currently available for melioidosis (Titball et al., 2017), which further intensifies concerns of a possible emerging public health threat.

Due to intrinsic resistance to a wide range of antibiotics, *B. pseudomallei* possesses a remarkable intrinsic array of genetic and virulence factors (Limmathurotsakul et al., 2016). Due to limited therapeutic options, carbapenems are considered the gold standard drug recommended for life-threatening sepsis cases. To date, all *B. pseudomallei* resistance mechanisms are conferred by chromosomal mutations. However, acquired resistance could be developed if the infection leads to prolonged sepsis and treatment failure (Sarovich et al., 2018). Studies of acquired resistance against β-lactam drugs, including carbapenems, identified three different phenotypic variations, mainly resulting from the *penA* gene, which is the most critical chromosomal gene and encodes a class A membrane-bound lipoprotein (Bugrysheva et al., 2017). Moreover, *omp38*, which encodes an outer membrane porin, is thought to contribute to ceftazidime and carbapenem resistance (Rhodes and Schweizer, 2016).

In China, the first case of melioidosis was reported in 1990 in Zhanjiang, Guangdong province. Subsequently, it was shown to be endemic in many tropical southern provinces, including Fujian, Guangxi, Guangdong, and Hainan, among which Hainan province has the most significant number of reported cases (Yang, 2000; Chen et al., 2015). In a retrospective study, between 1990 and 2005, a total of 44 melioidosis cases were detected in patients at a hospital in Zhanjiang, 25 of whom died (56.8%) (Zheng et al., 2019). The cases of melioidosis in Guangdong province are mainly concentrated in Zhanjiang city and the surrounding counties and villages, with new cases occurring every year, leading to occasional outbreaks. Over 40 years have passed since melioidosis was first reported in southern China. However, many aspects of this disease are still lacking, including the disease burden and its true epidemiology. Research on antibiotic resistance, genetic characterization, and the molecular epidemiology of pathogenic bacteria are also scarce. This study was conducted on eight strains of *B. pseudomallei* recovered from Guangdong province to understand the mechanisms for drug resistance and explore the genomic characterization. The comprehensive genomic characterization could further define the genetic basis of different resistance determinants, virulence factors, and mutation genes that potentially contribute to the pathogenicity of these strains.

## Materials and methods

### Sources of isolates

Eight strains of *B. pseudomallei* were collected from clinical specimens (blood and wound sepsis) of the sentinel hospitals of the pathogen identification network in Guangdong province in 2018–2019 (Table 1). All strains were stored at −70°C in Luria-Bertani broth containing 25% glycerol, until further identification and experiments.

**Table 1:** Number of *B. pseudomallei* strains recovered from Guangdong during 2018–19.

***B. pseudomallei* isolates**	**Date collected**	**Source**	**Location**	**Age**	**Gender**
BP-GD19BP04	12.04.2019	Clinical	Maoming Hospital	76	Male
BP-GD19BP05	12.09.2019	Clinical	Maoming Hospital	63	Male
BP-GD18BP02	04.26.2018	Clinical	Guangdong Medical University Hospital, Zhanjiang	53	Female
BP-GD18BP04	07.21.2018	Clinical	Lianjiang People’s Hospital, Zhanjiang	58	Female
BP-GD18BP06	08.31.2018	Clinical	Lianjiang People’s Hospital, Zhanjiang	51	Male
Bp-GD19BP01	06.15.2019	Clinical	Lianjiang People’s Hospital, Zhanjiang	missing	Male
BP-GD19BP02	08.31.2019	Clinical	Zhanjiang Guangdong Agricultural Reclamation Central Hospital	32	Male
BP-GD19BP03	07.22.2019	Clinical	Lianjiang People’s Hospital, Zhanjiang	missing	Male

### Main reagents and instruments

The following were used for experiments: Gram-negative bacteria drug sensitivity plates (products of the Shanghai Xingbai Company, China), bacterial genomic DNA extraction (QIAamp® DNA Mini Kit, Germany), and matrix-assisted laser desorption ionization-time of flight mass spectrometry (MALDI-TOF MS, Zybio EXS3000, China), Illumina NovaSeq sequencing platform (Illumina, USA) and the corresponding reagents.

### Strain identification and susceptibility testing

Prior to testing, each isolate was subcultured by steaking the thawed stock culture on trypticase soy agar (Oxoid Ltd., Basingstoke, United Kingdom) containing 5% horse blood and incubating for 48 h at 37°C. All isolates were screened phenotypically by Gram staining, latex agglutination (using latex particles coated with monoclonal antibodies specific for the 200-kDa exopolysaccharide of *B. pseudomallei*) (Duval et al., 2014), immunofluorescence assay (Olympus, Tokyo, Japan), and standard biochemical tests (arginine decarboxylase test, sulfide indole motility agar, triple sugar iron agar, citrate, and urea hydrolysis test). Isolates were further confirmed as *B. pseudomallei*, by the hospital labs, using matrix-assisted laser desorption ionization-time of flight mass spectrometry (MALDI-TOF MS).

All strains were streaked on Columbia agar containing 5% sheep blood to test for antibiotic susceptibility. Fresh new colonies of each strain were suspended in 0.45% saline to make turbidity equivalent to 0.5 McFarland standard (~1.5 × 10^7^ CFU/ml), and this solution was used for antibiotic susceptibility testing using the broth microdilution method. The quality control strain used was *Escherichia coli* ATCC 25922. The results of antibiotic susceptibility testing were interpreted according to the Clinical and Laboratory Standards Institute (CLSI) (Hindler and Richter, 2016).

### DNA extraction and whole-genome sequencing

Genomic DNA of *B. pseudomallei* strains (*n* = 8) were extracted from overnight cultures using a QIAamp DNA purification mini kit according to the manufacturer guidelines, and stored at −20°C. DNA library construction was performed using a NEBNext® Ultra DNA Library Prep Kit for Illumina (NEB, USA) and sequenced on an Illumina NovaSeq platform.

### Raw data pre-processing and whole-genome analysis

The quality of raw data from paired-end sequencing was checked using FastQC (v.0.11.6) (Brown et al., 2017). Fastp (v.0.23.2) was performed for the quality filtering to remove the low-quality reads, adapters, and polyG tails (Chen et al., 2018). Before and after filtering with Fastp, the quality was checked using FastQC to control the credibility and effectiveness. The whole genome paired-end reads were assembled using the genome assembly algorithm SPAdes (v.3.15.3) with default parameters (Bankevich et al., 2012). QUAST (v.5.0.2) was used to assess the quality of the genome assemblies (Gurevich et al., 2013). *B. pseudomallei* strain Mahidol-1106a (GenBank accession NZ_CP008781.1) was selected as a reference strain from NCBI for genome assembly and alignment for SNP identification. BWA-MEM (v.0.7.17) was used for mapping, and the file format was converted using SAMtools (v.1.9) (Li and Durbin, 2009; Li et al., 2009). After merging with SAMtools, Picard (v.2.6) was used to sort out and remove duplicates from the same data sets. Genome Analysis Toolkit (GATK v.4.2.5.0) was used for SNPs and indel identification (McKenna et al., 2010). The selection of SNPs in the bacterial genomes was based on the following parameters: (GATK SelectVariants -V gatk.vcf -O bp.snp.vcf --select-type-to-include SNP;Filter SNPs:GATK VariantFiltration -O bp.snp.fil.vcf. Temp -V bp.snp.vcf --filter-expression ‘QUAL <30.0 || QD < 2.0 || FS > 60.0 || SOR > 4.0’ --filter-name low --cluster-window-size 10 --cluster-size 3 --missing-values-evaluate-as-failing grep PASS bp.snp.fil.vcf.temp > bp.snp.fil.vcf).

### Genome annotation and identification of resistance and mobile element factors

Open reading frames (ORFs) were first predicted using Prodigal (v.2.6.2) (Hyatt et al., 2010). The ORF on contigs of each clean sample read was annotated in the comprehensive antibiotic resistance database (CARD) using Bowtie (v2-2.2.9) to find the abundance of resistance genes. Similar to the antibiotic resistance gene (ARG) analysis, the contigs of each sample were compared with the mobile genetic element (MGE) database. The MGE database is derived from the National Center for Biotechnology Information (NCBI) database and the PlasmidFinder database, which consists of 278 different gene types and more than 2,000 unique sequences (Pärnänen et al., 2018). The annotations were classified as MGEs based on string matches with the following keywords: insertion sequences for transposases, integrases, insertion sequence common regions (IS*CRs*), plasmids, Tn916 transposon, and tnp-ISCR transposases. Bowtie (v 2–2.2.9) platform was performed using (-D 20 - r3 -N 1 -L 20 -i S,1,0.50) parameters, for mapping contigs in the ARG and MGE databases.

### Multilocus sequence typing, virulence factors, and mutations

For MLST assignments of the eight *B. pseudomallei* isolates, we used the publicly available MLST server database (http://pubmlst.org/bpseudomallei/), which uses WGS data to identify bacterial sequence types (STs). The MLST alleles at each locus were analyzed for each strain. The closest matching MLST alleles were selected, and the STs were identified based on the combination of MLST alleles. To identify virulence factors, analysis was performed using the virulence factor database (VFDB) (http://www.mgc.ac.cn/VFs/main.htm). OncoPrint, one of the tools of cBioPortal (http://cbioportal.org), was used to identify mutations in different genes responsible for resistance in bacterial genomes, and also yielded a concise and compact graphical summary of genomic alterations (Gao et al., 2013).

### Phylogenomic analysis

Phylogenetic analysis of the *B. pseudomallei* strains was performed using kSNP3.0 (Gardner et al., 2015) to determine the genetic relationships among isolates. We constructed the phylogeny tree using KSNP (v3.021), found single nucleotide polymorphisms in DNA sequences using (−k 31 -ML -NJ -vcf -CPU 30 -core -min_frac 0.5) parameters. kSNP3.0 can automatically annotate the identified SNPs in the given genomes and estimates the phylogenetic trees by parsimony, neighbor-joining, and maximum likelihood, with various node labels, including the number of SNPs specific to each node. In this study, phylogenetic analysis was based on 130 *B. pseudomallei* genomes, derived from the eight genomes from this study together with 122 *B. pseudomallei* genomes from other regions. The details of all genomes used in this study are listed in Supplementary Table S1. The tree was visualized and annotated using ChiPlot (https://www.chiplot.online/).

## Results

### Antibiotic susceptibility testing

Antibiotic susceptibility testing revealed that all eight *B. pseudomallei* strains obtained in this study were sensitive to tetracycline, trimethoprim-sulfamethoxazole, ceftazidime, imipenem, amoxicillin-clavulanate, and doxycycline. The antibiotic susceptibility profiles of each strain are listed in Table 2.

**Table 2:** Minimum inhibitory concentrations (MICs) of the eight clinical *B. pseudomallei* isolates.

**Sample ID**	**TET** **(μg/mL)**	**TMP/SMX** **(μg/mL)**	**CAZ** **(μg/mL)**	**IMP** **(μg/mL)**	**AMC** **(μg/mL)**	**DOX** **(μg/mL)**
BP-GD19BP04	2	≤0.25/4.75	4	≤0.25	4/2	≤0.5
BP-GD19BP05	4	≤0.25/4.75	2	≤0.25	4/2	≤0.5
BP-GD18BP02	4	0.5/9.5	1	≤0.25	4/2	≤0.5
BP-GD18BP04	2	0.5/9.5	1	≤0.25	4/2	≤0.5
BP-GD18BP06	2	≤0.25/4.75	2	≤0.25	4/2	≤0.5
Bp-GD19BP01	4	1/19	1	≤0.25	4/2	≤0.5
BP-GD19BP02	4	1/19	2	≤0.25	4/2	≤0.5
BP-GD19BP03	4	≤0.25/4.75	2	≤0.25	8/4	≤0.5

CLSI MIC breakpoints: TET: tetracycline (S ≤ 2; R ≥ 16), TMP/SMX: trimethoprim/sulfamethoxazole (S ≤ 2/38; R ≥ 4/76), CAZ: ceftazidime (S ≤ 8; R ≥ 32), IMP: imipenem (S ≤ 4; R ≥ 16), AMC: amoxicillin/clavulanate (S ≤ 8/4; R ≥ 32/16), DOX: doxycycline (S ≤ 4; R ≥ 16).

### Genomic features of *Burkholderia pseudomallei* draft genomes

The draft genomes of eight *B. pseudomallei* in this study varied in size (7,090,270 bp, 7,006,915 bp, 7,004,666 bp, 7,062,584 bp, 7,222,130 bp, 7,124,471 bp, 7,239,821 bp, and 7,296,469 bp), with a GC content (number of guanine and cytosine sites of the same strand divided by total DNA sequence length) of ~68% (Table 3). We assembled a total of 3,415 contigs, with an average of 427 contigs per sample. The total number of SNPs identified in each sample were: GD18BP02 = 14,666, GD18BP04 = 13,389, GD18BP06 = 17,674, GD19BP01 = 18,577, GD19BP02 = 14,894, GD19BP03 = 16,777, GD19BP04 = 20,170 and GD19BP05 has 19,621 SNPs are listed in Supplementary Table S2.

**Table 3:** Genomic features of the eight *B. pseudomallei* draft genomes.

**Strain**	**18BP02**	**18BP04**	**18BP06**	**19BP01**	**19BP02**	**19BP03**	**19BP04**	**19BP05**
**Genome size (bp)**	7,090,270	7,006,915	7,004,666	7,062,584	7,222,130	7,124,471	7,239,821	7,296,469
**GC content (%)**	68.22	68.26	68.27	68.19	68.14	68.2	67.99	67.96
**N50**	92,341	58,697	73,535	102,767	73,240	92,513	102,360	103,494
**L50**	26	39	32	23	31	25	25	23
**Contigs**	173	241	207	138	187	177	170	158

### Antimicrobial resistance genes and mobile genetic elements

**Figure 1:**
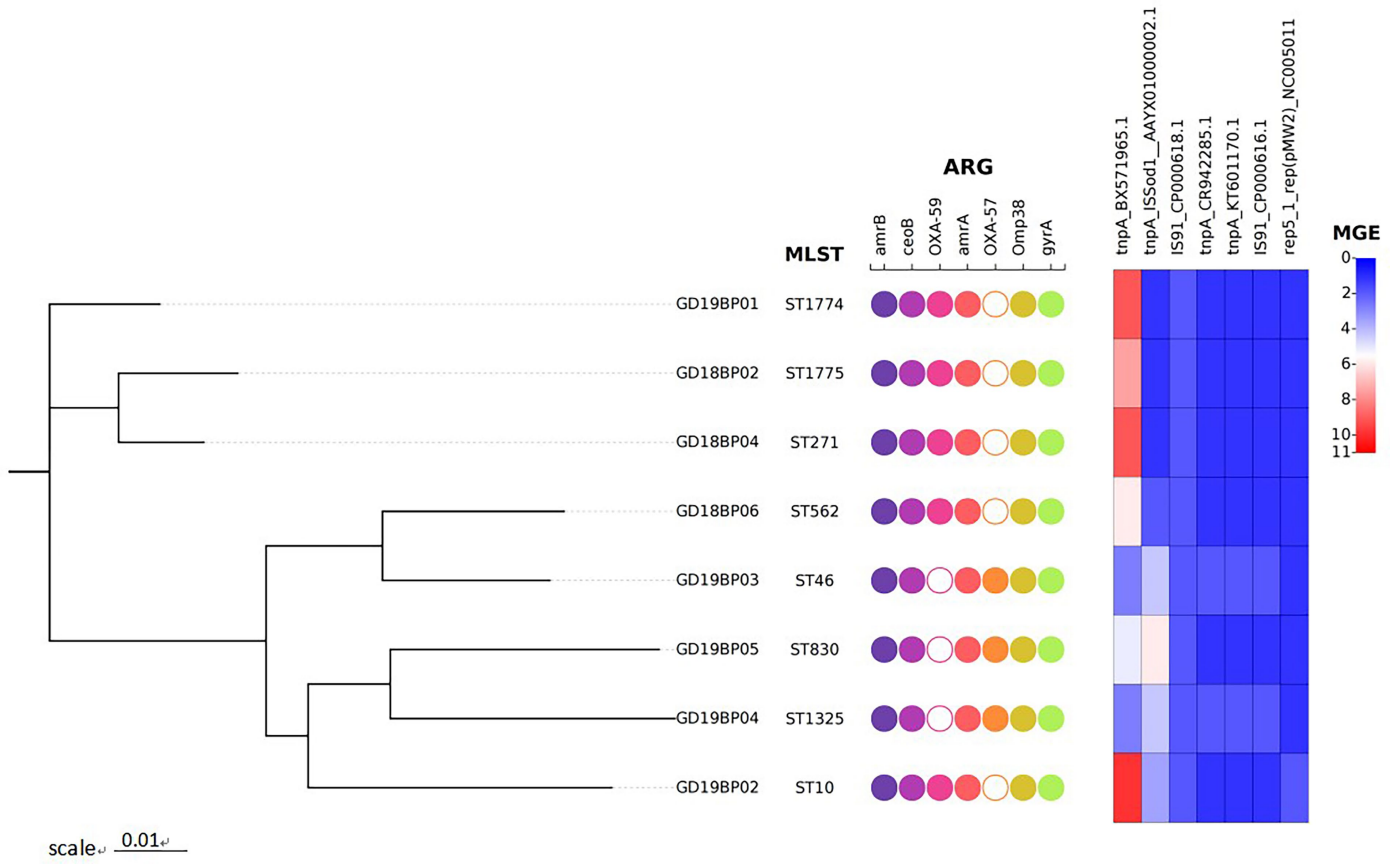
Phylogenetic tree of the eight *B. pseudomallei* strains recovered from Guangdong, China. The figure represents the genetic features of these strains. Circles in the figure are binary plots, with filled colors indicating that these isolates were positive for the genetic features mentioned on the top, while hollow white circles indicate that the corresponding genes are missing in that sample. The heatmap on the right shows the number of mobile genetic elements (MGEs).

The ARGs and MGEs identified in each *B. pseudomallei* are presented in Figure 1. All eight strains of *B. pseudomallei* were positive for *amrA*, *amrB*, *omp38*, *gyrA*, and *ceoB*. The *bla*_OXA-59_ was detected in five strains, while three strains were positive for *bla*_OXA-57_. The *omp38* is the outer bacterial membrane porin responsible for reducing the permeability against beta-lactams. Five strains were positive for the presence of the IS*91* family, while two were positive for the *tnpA* transposase gene.

### Multilocus sequence typing, virulence factors and mutation genes

**Figure 2:**
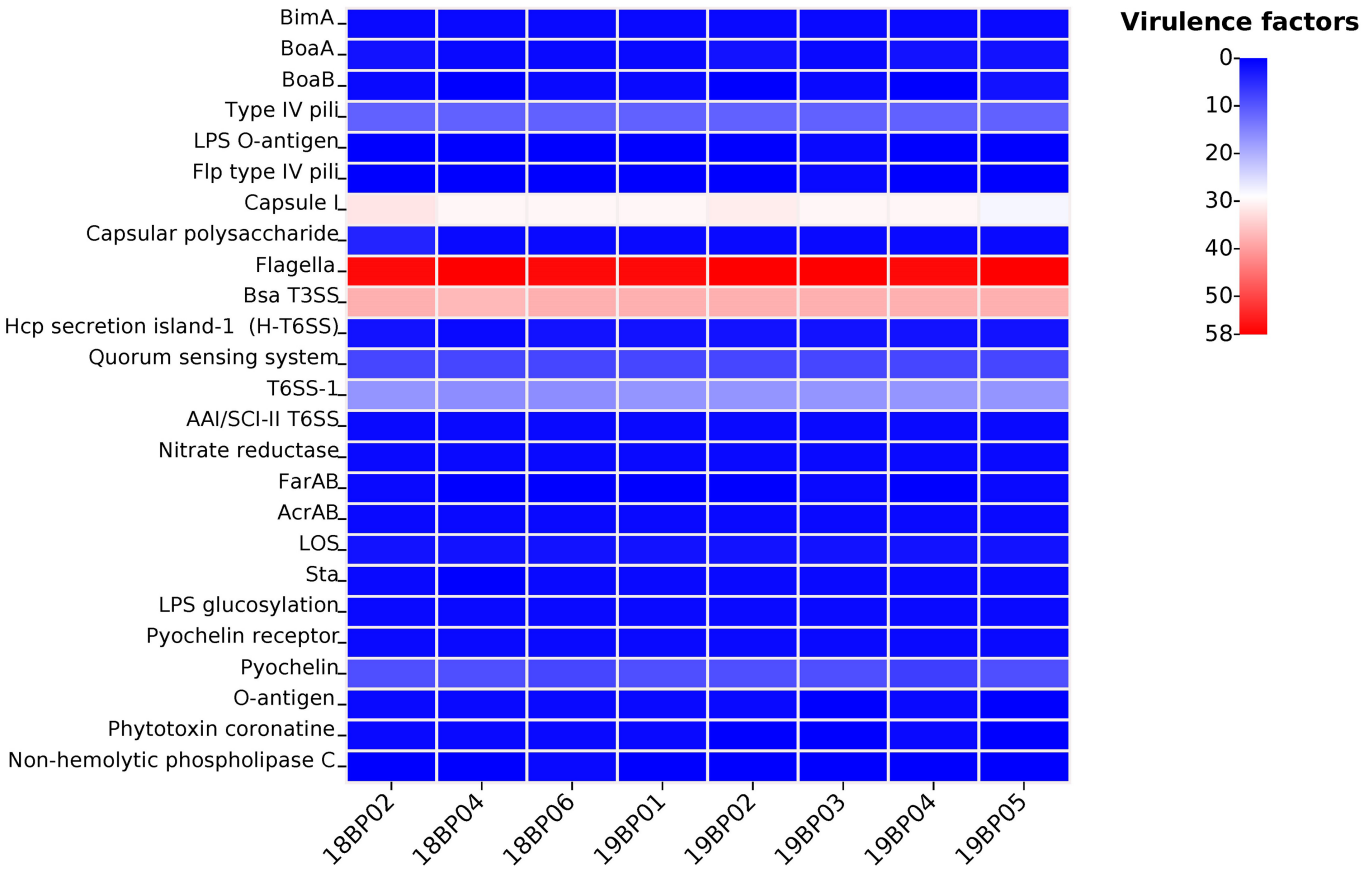
Heat map displaying virulence genes distribution in the eight *B. pseudomallei* strains. Each isolate has a different number of virulence factors (*n =* 0–58).

**Figure 3:**
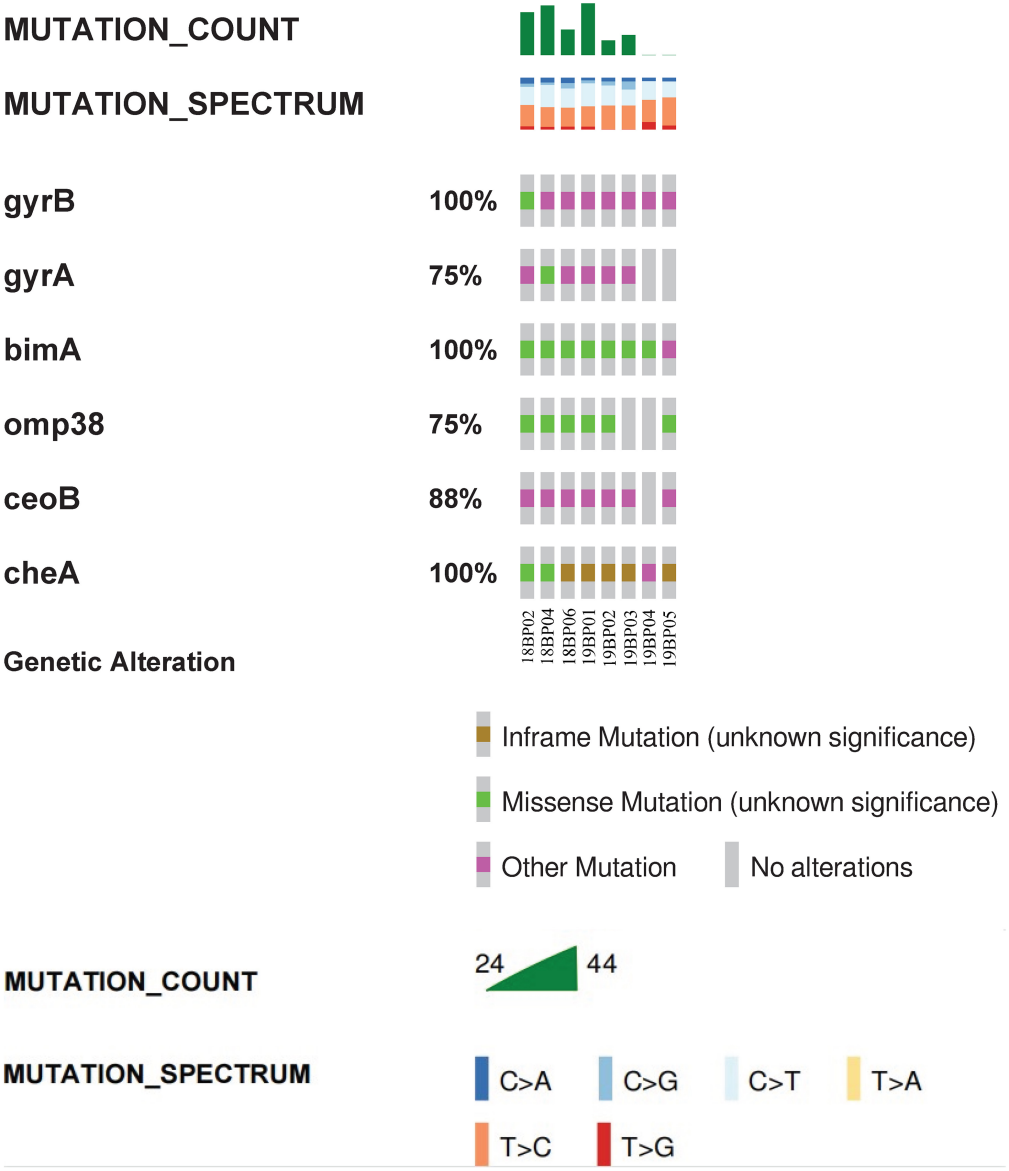
Visual summary of *bimA*, *ceoB*, *cheA*, *gyrA*, *gyrB* and *omp38*. The OncoPrint tool of cBioPortal summarizes genomic alterations in all queried genes across a sample set. Each row represents a gene, and each column represents a sample. The green bars indicate missense mutations, brown bars indicate inframe mutations, and purple squares indicate unknown types of mutations. Mutation count is the total number of mutations for the six genes in the sample, while the different colors represents the different types of base changes.

Whole-genome MLST analysis of the *B. pseudomallei* strains identified eight different sequence types (STs): ST1774, ST1775, ST271, ST562, ST46, ST830, ST1325, and ST10 (Figure 1). To explore virulence determinants in the current study, we mapped the assembled genomes of the 8 strains against the core dataset of virulence factors in VDFB. We identified a total of 165 different virulence genes (Supplementary Table S3), including those encoding flagellum-related gene cluster, capsular polysaccharide, multiple specialized secretion systems (T3SS, T4SS, and T6SS), and a diverse complement of autotransporters (*bimA*, *boaA*, *boaB*) (Figure 2). We used OncoPrint to find the SNPs, affecting *bimA, ceoB, cheA, gyrA, gyrB,* and *omp38*, in the eight samples of *B. pseudomallei* in this study. From OncoPrint, mutations were detected in only three genes, i.e., *gyrB*, *bimA*, and *cheA*, but the types of mutations were different: a previously uncharacterized mutation was found in *gyrB*, and missense mutations were found in *bimA*, while an in-frame insertion was found in *cheA* (Figure 3).

### Phylogenetic analysis

**Figure 4:**
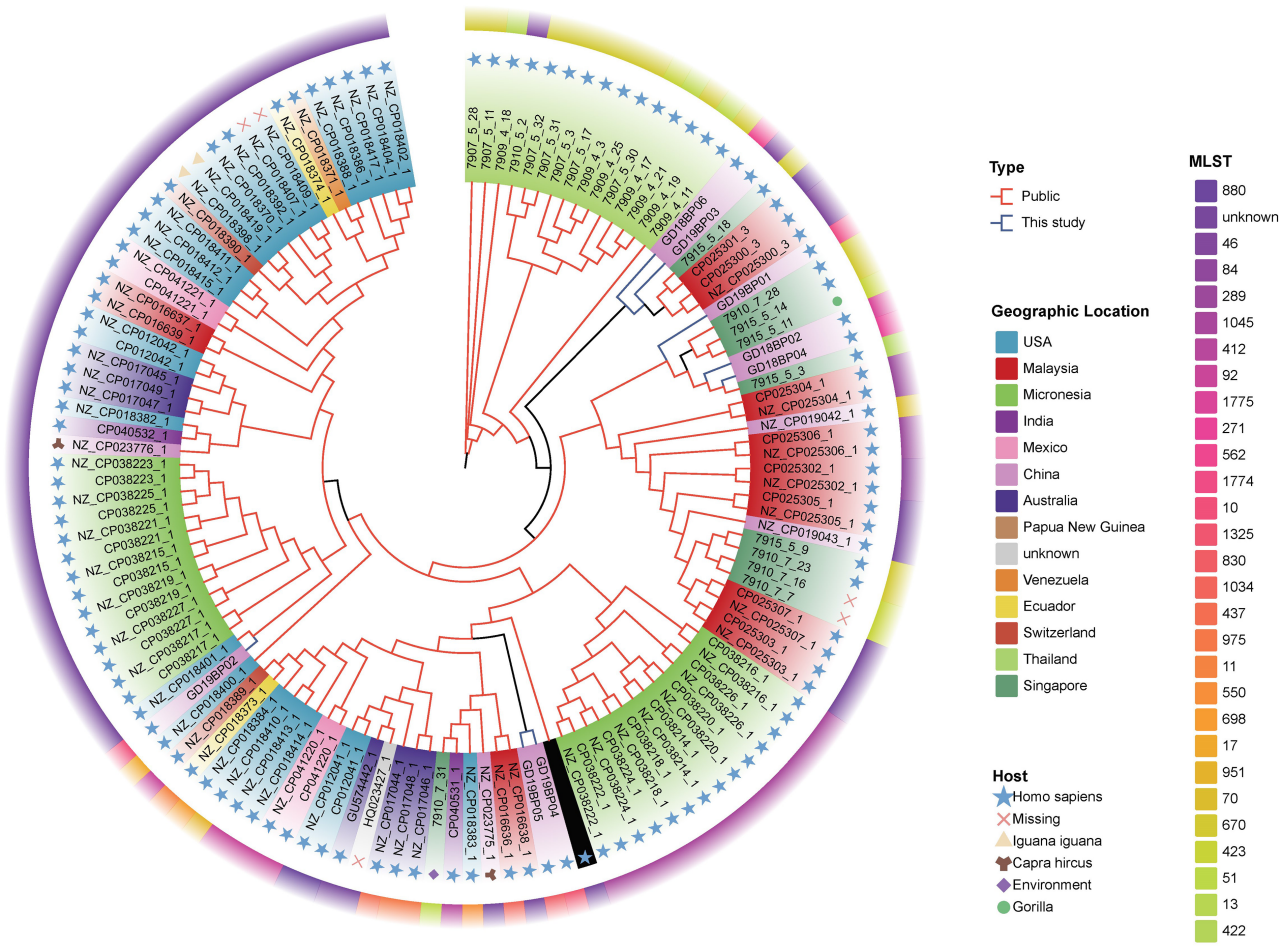
Phylogenomic comparison of Guangdong *B. pseudomallei* strains (*n* = 8) with global *B. pseudomallei* reference strains (*n* = 122). The maximum-parsimony phylogenetic tree was constructed from 119 *B. pseudomallei* genome sequences, including the eight genomes from this study. The inner circle with varying colors represents different countries. The colors in the middle circle show the MLST of each genomic isolate, while the outermost circle with different symbols displays the strain sources.

Phylogenetic analysis of *B. pseudomallei* was conducted, focusing on human clinical and animal origin isolates from different geographical regions. Comparative analysis was based on eight assembled genomes of *B. pseudomallei* from this study together with a global set of 122 genomes from the public databases to study the relationships among the genomic strains. The parsimony tree had different clades displaying clear divergence of global and local strains from this study according to the different regions (Figure 4). Different clades indicated that the *B. pseudomallei* has a high genetic diversity. The isolates from this study (blue branches) located in different clades with different branches. Two strains, GD19BP04 and GD19BP05, had high similarities and were located on the same branch, while GD19P01 and GD19P02 were positioned on a different branch and had a proximity relationship with isolates previously reported from Guangdong, China. Three other strains, GD18BP02, GD18BP04, and GD19BP01, were clustered within one clade closely related to strains previously reported from China. The remaining two strains, GD18BP06 and GD19P03, located on the same clade and sharing nodes with clusters, were similar to isolates from China, Malaysia, and Singapore.

## Discussion

*B. pseudomallei* is a cause of melioidosis, which leads to 10 to 40% fatal illness due to acquired pneumonia and septicemia in endemic countries (Limmathurotsakul et al., 2016). *B. pseudomallei* was previously known to be highly endemic in Southeast Asia and Northern Australia, but is now emerging globally (Limmathurotsakul et al., 2016). Melioidosis has diverse clinical manifestations, and patients with diabetes mellitus and renal and respiratory problems are at higher risk of acquiring the disease (Wiersinga et al., 2018). The current study report showed that the melioidosis is not only limited to the endemic areas but also sporadically appeared in non-endemic regions of mainland China (Zheng et al., 2019).

The antibiotic susceptibility of *B. pseudomallei* strains in this study was tested against six antimicrobial agents, i.e., amoxicillin-clavulanate, ceftazidime, doxycycline, imipenem, tetracycline, and trimethoprim-sulfamethoxazole, which are most frequently used in treating melioidosis (Dance, 2014; Sullivan et al., 2020). All of the recovered strains in this study were sensitive to these antimicrobial agents. *B. pseudomallei* isolates collected prior to antibiotic treatment are generally susceptible to the commonly used clinical antimicrobials including, ceftazidime, doxycycline, imipenem, meropenem, amoxicillin-clavulanate and trimethoprim-sulfamethoxazole (Madden et al., 2021). Two class D β-lactamases (oxacillinases named *bla*_OXA-57_ and *bla*_OXA-58_) were identified, that is ubiquitous to *B. pseudomallei* strains, and have been reported in other global *B. pseudomallei* strains (Niumsup and Wuthiekanun, 2002; Keith et al., 2005). However, no oxacillinase has yet been shown to cause resistance to ceftazidime in clinical *B. pseudomallei* isolates (Schweizer, 2012; Zheng et al., 2021b), and the resistance to ceftazidime in clinical *B. pseudomallei* isolates is caused by the mutations within the class A β-lactamase PenA (Rholl et al., 2011; Schweizer, 2012; Zheng et al., 2021b).

We identified *amrA* and *amrB* genes, which are the main components of the AmrAB-OprA multi-drug efflux pump mechanism (Podnecky et al., 2015). Three efflux pumps, i.e., AmrAB-OprA, BpeAB-OprB, and BpeEF-OprC, make the *B. pseudomallei* multi-drug resistant to antibiotics through alterations in their own regulators; *AmrR, BpeR,* and *BpeT* and *BpeS* (Jassem et al., 2014; Podnecky et al., 2015). However, we did not find any of these genes responsible for point mutations previously reported in Australian and Hainan genomes (Webb et al., 2020a; Zheng et al., 2021a). We also found th*e omp38* gene, which encodes an outer membrane porin, present in every *B. pseudomallei* isolate, and has no relevance to clinically relevant antimicrobial resistance (Aunkham et al., 2014; Rhodes and Schweizer, 2016; Zheng et al., 2021b). All strains were positive for a putative cytoplasmic membrane component of the multi-drug efflux system (*CeoB*) gene, which confers resistance to ciprofloxacin, chloramphenicol, and trimethoprim, first described in *B. cenocepacia* (Nair et al., 2004). *CeoB* is a part of the RND family (CeoAB-OpcM), which is parallel to *B. pseudomallei*. However, the individual efflux pump drug sensitivity patterns, bestowed by the individual efflux pumps, are somewhat dissimilar (Podnecky et al., 2015). In the present study, the fluoroquinolones resistance gene *gyrA* was detected in all the *B. pseudomallei* strains involved in developing resistance in different microorganisms (Podnecky et al., 2015). Several studies have reported that mutations in *gyrA* are responsible for developing quinolone resistance (Viktorov et al., 2008; Rhodes and Schweizer, 2016).

We identified 165 types of virulence factors, including those carrying different complements of autotransporters (*bimA*, *boaA*, *boaB,* etc.), capsular polysaccharide I (CPS), multiple secretion systems (T3SS, T6SS, etc.), and flagellum-related factors, with 100% identity to the previous report from Hainan (Zheng et al., 2021a). Our results show the presence of virulence factors in *B. pseudomallei* strains, in China that have been reported in other global *B. pseudomallei* strains (Sarovich et al., 2014; Zheng et al., 2021a). *Burkholderia* intercellular factor A (*BimA*), which is responsible for actin polymerization by *B. pseudomallei* (Gassiep et al., 2020), was identified in our isolates. *BimA* is typically found in *B. pseudomallei* across Asia and has been linked with pneumonia (Sarovich et al., 2014). We did not find any *BimA* variant, i.e., *BimA*_Bm,_ which has only been reported in Australian and Indian isolates and is associated with neurological disease (Mukhopadhyay et al., 2011; Sarovich et al., 2014; Webb et al., 2019). Until now, no *BimA*_Bm_ variant has been observed in clinical and environmental isolates from Southeast Asia, which could explain the low incidence of encephalomyelitis in this region (Sarovich et al., 2014). Two other virulence genes, *boaA* and *boaB,* have been variably observed in *B. pseudomallei* strains in China and globally. The BoaA protein shares resemblance with the YadA adhesin of *Yersinia enterocolitica*, and was found in 100% of our isolates (Balder et al., 2010; Adler, 2011). A similar gene, *boaB*, which is specific to *B. pseudomallei*, has been reported to be in ~33% in global strains and ~ 12–56% in the strains from the Hainan province (Zheng et al., 2021a), while here, we observed *boaB* in 75% of the isolates. Another virulence gene *chbp,* that encodes a key secretion effector for the T3SS was identified in 100% of our isolates, and has been previously observed in 89% of the Hainan strains and ~ 72% of global isolates (Zheng et al., 2021a). This study detected filamentous hemagglutinin gene surface proteins (*flhA* and *flhB*), that act as adhesins. However, no *flhAB*3 variant was found, which has been associated with localized skin abscess previously reported in Australia, India, Thailand, and Myanmar isolates (Sarovich et al., 2014; Shaw et al., 2019; Webb et al., 2020b).

*B. pseudomallei* was previously considered to be limited only to tropical regions. Nevertheless, it is rising and being reported in other geographical locations (Fisher and Harris, 2014). Multi-locus sequence typing (MLST) analysis has been commonly used for molecular and epidemiological characterization of melioidosis in various regions. Recent epidemiological studies from Hainan province in China characterized 102 and 52 *B. pseudomallei* strains and found 63 sequence types (STs), among which ST46, ST50, ST54, ST58, and ST70 were predominant (35%) (Fang et al., 2016, 2019). Here, we characterized eight *B. pseudomallei* strains belonging to 8 ST types, among which ST271, ST562, ST46, and ST1325 have been reported previously in Hainan province (Chen et al., 2015; Chen and Li, 2018; Fang et al., 2019; Zheng et al., 2021a), with ST10 also have been reported from Malaysia (Zueter et al., 2018), and ST830 have been reported from Australia, Thailand, and Cambodia. In this study, two new sequence types, ST1774 and ST1775, were found with no other matches in *B. pseudomallei* database. The emergence of new genotypes suggests that *B. pseudomallei* is a robust pathogen that can persist in diverse geographical regions and have the ability to generate new subtypes (Webb et al., 2020a). An epidemiological study from India showed that 30 strains (93.7%), from 32 *B. pseudomallei* isolates had novel allelic profiles (Tellapragada et al., 2016). Another study from Malaysia found 32 different STs, of which 40% were new and had never been reported (Zueter et al., 2018). It can be understood that *B. pseudomallei* has specific geographical and regional genotyping advantages. However, within the same region, novelty and variations exist among the genotypes, illustrating strong genomic plasticity, leading to genetic diversity. Whole-genome sequencing (WGS) analysis compared with MLST is more consistent and effective in tracing the epidemiological linkages among *B. pseudomallei* strains of melioidosis. Price et al. recovered 12 *B. pseudomallei* isolates from a chronic melioidosis patient over a 32-month period, and found similar multi-locus sequence types (STs) (Price et al., 2015). However, by WGS, they found polyclonal infection, making it the first study to find mixed infection with the same sequence type (ST). Sarovich et al. used large-scale comparative genomics to investigate the exact origin of melioidosis in Africa, and found that the *B. pseudomallei* strains likely originated in Asia and the ancestors are linked to the South American strains suggesting long term endemicity in the region (Sarovich et al., 2016). The phylogenetic evolution of the isolates in this study was constructed based on genome-wide SNP typing (WGST). The results of the tree showed that the 8 strains of *B. pseudomallei* share different nodes and branches, which further indicates that the melioidosis in this area results from a high genetic diversity of *B. pseudomallei*, and at the same time, the *B. pseudomallei* are closely linked to the isolates from other regions. Two of the newly found STs, i.e., ST1774 and ST1775 are closely related to ST289 which has been reported in Malaysia. It is speculated that the new genotypes are likely to originate from the epidemic in Malaysia, and have similar clonal lines or have a common evolutionary ancestor. However, extensive allelic variations were observed among the three isolates, revealing higher genomic recombination rates in the *B. pseudomallei.*

We acknowledge several limitations in this study. First, we recovered limited samples, which was only a snapshot of the entire region. Data was missing concerning patient clinical history, such as the severity of the disease (bacteremia, septic shock), antibiotics used, clinical manifestations, and recovery/death, but the majority of the strains were sensitive to commonly used antibiotics. Further surveillance studies on a large scale should be performed from these regions, along with 3^rd^ generation sequencing platforms, which will give us full insight into the genomic features of *B. pseudomallei.*

## Conclusion

The present study reports the identification of different mutation and virulence factors which are responsible for resistance development and associated with different pathological and neurological disorders. Two new sequence types, ST1774 and ST1775, were found for the first time with no other matches in *B. pseudomallei* database. Phylogenomic analysis revealed that the melioidosis in this area results from a high genetic diversity and linking to *B. pseudomallei* isolates from other regions. Further surveillance studies on a large scale should be performed from these regions to better understand the genomic diversity of the strains and prevent the sporadic cases, in both endemic and non-endemic areas.

## Data availability statement

The sequence data mentioned in the present study were deposited into the GenBank NCBI database under the BioProject. PRJNA857175 with accession numbers SRR20747241, SRR20747242, SRR20747243, SRR20747244, SRR20747245, SRR20747246, SRR20747247 and SRR20747248.

## Ethics statement

Ethical approval was provided by the Human Research Ethics Committee of Shantou University Medical College (SUMC-2021-51). Consent forms from the patients were waived by the ethical committee as all the clinical samples were obtained from the hospital laboratory.

## Author contributions

MS, XL, and MZ analyzed the data. BK and DH conducted the experiments. YY helped with the analysis. MS wrote the original manuscript. XD and XJ reviewed the manuscript. All authors contributed to the article and approved the submitted version.

## Funding

This study has been graciously supported by as follows:

1. Establishment of key techniques and reference library for standardization identification of pathogens of important vector-borne diseases in South China (project number: 2018ZX10734404)
2. Guangdong Provincial Center for Disease Control and Prevention, Guangdong Workstation for Emerging Infectious Disease Control and Prevention
3. The 2020 Li Ka Shing Foundation Cross-Disciplinary Research Grant (project number: 2020LKSFG03E).
4. The National Natural Science Foundation of China for International Young Scientists (No. 42150410383)

## Conflict of interest

The authors declare that the research was conducted in the absence of any commercial or financial relationships that could be construed as a potential conflict of interest.

## Publisher’s note

All claims expressed in this article are solely those of the authors and do not necessarily represent those of their affiliated organizations, or those of the publisher, the editors and the reviewers. Any product that may be evaluated in this article, or claim that may be made by its manufacturer, is not guaranteed or endorsed by the publisher.

## Supplementary material

The Supplementary material for this article can be found online at: https://www.frontiersin.org/articles/10.3389/fmicb.2022.980525/full#supplementary-material

SUPPLEMENTARY TABLE S1 Details of all genomes used in this study.

SUPPLEMENTARY TABLE S2 Details of all mutations and SNPs found in *B. pseudomallei* strains from this study.

SUPPLEMENTARY TABLE S3 Virulence factors identified in *B. pseudomallei* strains from this study.
