# Supplementary material for: Genomic diversity of resistant and virulent factors of Burkholderia pseudomallei clinical strains recovered from Guangdong using whole genome sequencing
Source: Front Microbiol. 2022 Oct 28;13:980525. doi: 10.3389/fmicb.2022.980525 (PMC9649843; doi:10.3389/fmicb.2022.980525)
Supplement: Supplementary file 4 [file Table_4.DOCX]

**Supplementary Table 4. BioSample IDs for genomes, TaxID IDs, and Sample IDs of this study**

|  | | |
| --- | --- | --- |
| Sample ID | BioSample ID | TaxID |
| 18BP02 | SAMN29620754 | 2969396 |
| 18BP04 | SAMN29620755 | 2969397 |
| 18BP06 | SAMN29620756 | 2969398 |
| 19BP01 | SAMN29620757 | 2969399 |
| 19BP02 | SAMN29620758 | 2969400 |
| 19BP03 | SAMN29620759 | 2969401 |
| 19BP04 | SAMN29620760 | 2969402 |
| 19BP05 | SAMN29620761 | 2969403 |
